# Editorial: Current Advances in the Research of RNA Regulatory Enzymes

**DOI:** 10.3389/fgene.2019.00973

**Published:** 2019-10-09

**Authors:** Akio Kanai, Tohru Yoshihisa

**Affiliations:** ^1^Institute for Advanced Biosciences, Keio University, Tsuruoka, Japan; ^2^Faculty of Environment and Information Studies, Keio University, Fujisawa, Japan; ^3^Graduate School of Life Science, University of Hyogo, Ako-gun, Japan

**Keywords:** RNA regulatory enzyme, RNA-Binding Protein, RNA modification, transcription, translation

Almost 30 years ago, when the editors were graduate course students in life sciences in Tokyo, Japan, the importance of biochemistry was stronger than it is today, and it was necessary to understand fields such as protein purification and enzyme kinetics just to read a molecular biology paper. We remember a book that was very useful back then, entitled *The Biochemistry of the Nucleic Acids, 10th Edition* (Roger et al., 1986), which summarized a variety of enzymes related to the regulation of nucleic acids and their biological functions.

Because we have walked the paths of the life sciences with this experience of biochemistry, it is no exaggeration to say that we feel compelled to act as editors of the research topic ‘Current Advances in the Research of RNA Regulatory Enzymes’ in the RNA section of *Frontiers in Genetics*. Since our student days, huge attention has been drawn to the numerous noncoding RNAs, most of which were first identified at the beginning of this century. However, as you will have noticed already, only a few RNAs, such as antisense RNAs and ribozymes, exhibit regulatory functions by themselves *in vivo* while enzymes and protein factors are required for most scenarios related to almost all other noncoding RNAs. For example, for microRNAs (miRNAs) to function, a protein partner complex, called the ‘RNA-induced silencing complex’ (RISC), is essential, in which the Argonaute is the core protein. Many enzymes and protein partners are also required to construct a mature and functional miRNA after the precursor miRNA gene is expressed in the nucleus.

Although many RNA researchers are already aware of the importance of enzymes and other proteins in RNA Biology, the research efforts in this field are still heavily weighted towards the RNA side rather than toward the enzyme and protein side. Therefore, we set the editorial direction of our research topic towards the enzyme/protein side. Although we cannot list these enzymes as exhaustively as the biochemistry book mentioned above, we have collected 11 excellent review papers on this research topic. Many of them are from the field of tRNA research because we both work in this particular field. This is a pertinent direction because several important subjects, including RNA modification, RNA editing, intracellular RNA trafficking, and the regulation of RNA processing, can be exemplified in one type of RNA molecules, tRNA. Therefore, this e-book is focused on important concepts that hold true even if tRNA is replaced with another RNA molecule. The first half of the book considers enzymatic catalysis reactions in which RNAs are the substrates, and the second half considers RNA-binding proteins that control the fates of their target RNAs.

We begin with enzymes that modify the RNA termini. Tomita and Liu introduce a unique enzyme that forms a methyl cap on the 5′-monophosphate of tRNA^His^, and Yashiro and Tomita review the structure–function relationships of the uridyltransferases that act on the 3′-termini of noncoding RNAs, such as pre-miRNAs. Interestingly, the TUT4/7 uridyltransferase alternates between a protective or degradative role for the *let-7* miRNA precursor according to the presence or absence of its the partner protein Lin28, respectively. Shigematsu et al. describe how the 2′-3′-cyclic-phosphate-forming RNases create a novel ‘hidden’ layer of the transcriptome. We then move on to enzymes that modify internal nucleotides, especially in tRNAs. Hori summarizes various tRNA-modification enzymes in thermophilic bacteria and their regulation. He explains the importance of the network of modified nucleotides to the stability of tRNA at high temperatures. Dixit et al. focus on the multimerization of the tRNA methyltransferase subunits to accommodate various tRNA substrates, and the interdependent actions of methyltransferase and deaminase. These modifications at or near the anticodon greatly affect decoding of a near-cognate codon corresponding to the same amino acid by a particular tRNA. Hou et al. explain the effects of m^1^G37, next to the anticodon, on codon-specific translation and its utilization in Mg^2+^ homeostasis in bacteria. Some tRNAs are encoded by intron-containing genes, and their splicing is essential for their function. Hirata reviews the structural characteristics and evolutionary relationships of various types of archaeal tRNA-splicing endonucleases. Hopper and Nostramo describe the processing steps for eukaryotic small noncoding RNAs, especially tRNAs, ranging from terminal processing to tRNA-type splicing.

In the second half of the e-book, the proteins that interact with RNAs and their involvement in gene expression are examined. Important aspects here are the dynamics of intracellular movement and localisation of eukaryotic RNAs in addition to their funcational regulation. Hopper and Nostramo summarize how tRNAs move across the nuclear pore during their biogenesis in yeast, emphasizing the involvement of parallel pathways that mainly transport other classes of RNAs. We then address issues related to the transcription and translation of mRNAs, mainstream topics of RNA biology. First, Yokoyama reports that SL1, a subunit of the RNA polymerase I preinitiation complex for rRNA transcription, plays another role as a component of the super elongator complex required for transcription by RNA polymerase II, which is also related to the *MLL* oncogene. Beyond transcriptional control, mRNA translation itself provides another platform for regulation. Otsuka et al. describe recent progress in our understanding of the translational regulation by RNA-binding proteins, which recognize the AU-rich elements (ARE) that occur in many mRNAs. Some ARE-binding proteins regulate mRNA stability, whereas others control the initiation of translation. Organisms face various process malfunctions, and so they are equipped with sophisticated quality control systems to cope with such failures. These quality control mechanisms also function during translation. Ikeuchi et al. discuss the quality control mechanism induced by ribosomes stalled on mRNAs, and provide mechanistic insights into the ribosome-associated quality control that rescues stalled ribosomes on aberrant mRNA, preventing the production of unwanted proteins.

We discuss some RNA-related enzymes and RNA-interacting proteins in this e-book, retaining a focus on biochemical aspects. The proteins discussed above act on a limited but diverse set of target RNAs in their biogenesis, movement, function, and regulation, but we still do not have a full substrate list for each enzyme or protein. We hope that the issues discussed in this e-book have some bearing on the scientific interests of individual readers. We will be greatly pleased if this collection of review articles provides a useful guide to novel research directions.

## Author Contributions

AK and TY wrote the manuscript.

## Funding

This work was supported in part by a JSPS KAKENHI Grant-in-Aid for Scientific Research (C) (grant number 17K07517) and by a JSPS KAKENHI Gran-in-Aid for Scientific Research on Innovative Areas “Hadean Bioscience” (grant number 26106003) (to A.K.), as well as by a JSPS KAKENHI Grant-in-Aids for Scientific Research (C) (grant number 17KT0113 & 17K07289) and by a JSPS KAKENHI Gran-in-Aid for Scientific Research on Innovative Areas “Nascent Chain Biology” (grant number 17H05672) (to T. Y.).

## Conflict of Interest

The authors declare that the research was conducted in the absence of any commercial or financial relationships that could be construed as a potential conflict of interest.
